# 1497. Cryptococcal Antigen Screening and Pre-emptive Fluconazole Therapy in HIV-infected Patients: A Multicenter, Retrospective Study in China

**DOI:** 10.1093/ofid/ofad500.1332

**Published:** 2023-11-27

**Authors:** Yanqiu Lu, Yaokai Chen

**Affiliations:** Chongqing Public Health Medical Center, chongqing, Chongqing, China; Chongqing Public Health Medical Center, chongqing, Chongqing, China

## Abstract

**Background:**

The implementation and effectivention of CrAg screening programs and pre-emptive treatment remains unclear in China.

**Methods:**

We performed a multicenter, retrospective study at eight hospitals from 1st Jan 2019 to 31st Dec 2020, to evaluate whether CrAg screening programs and preemptive treatment are beneficial for saving lives in China.

**Results:**

A total of 12768 PLWH were screened for serum CrAg. The prevalence of serum CrAg positivity was 5.3% (678/12768). Lumbar puncture (LP) records were completed and available in 580 patients. Among them, 36.4% (369/580) were diagnosed with CM, and 63.6% (211/580) were diagnosed with isolated cryptococcal antigenemia (ICA). 123 HIV/ICA patients have available clinical data. Of these patients, a median CD4^+^ T-cell counts were 41cells/µL (IQR, 19-83). Upon Cox proportional-hazards regression analysis, patients with no preemptive fluconazole treatment had a threefold higher risk of CM and/or death [19.0% (8/42) vs 8.6% (7/81), *p*=0.037] compared to the preemptive fluconazole treatment participants. However, there was no statistically significant difference for the development of CM and/or death in different dosage (therapy as per WHO recommendations vs oral fluconazole 400mg daily therapy) of fluconazole therapy (*p*=0.836). Also, we found no statistically significant difference in the proportion of CrAg negativity [60.0% (12/20) vs 54.5% (18/33), *p*=0.779] and the median time from positivity to negativity of serum CrAg [14 (IOR:9.25, 14) vs 14 (10.5, 15), *p*=1.000] within one year follow up. In our study, Patients who with virological suppression had a lower incidence of CM and/or death than patients who with virological non-suppression [5.6% (1/18) vs (17.2% (5/29), *p*=0.384], however, there was no statistically significant difference in Cox proportional-hazards regression analysis.

**Figure:**
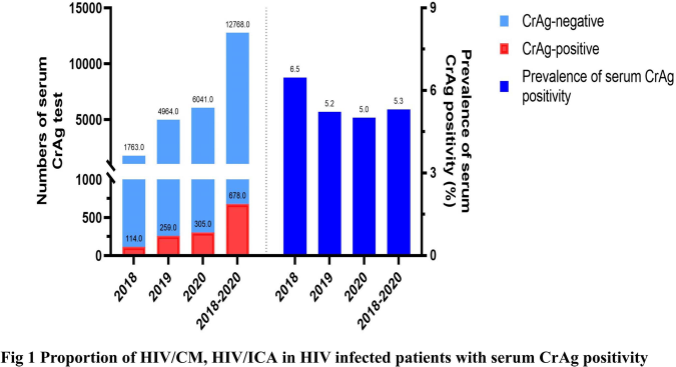

**Figure:**
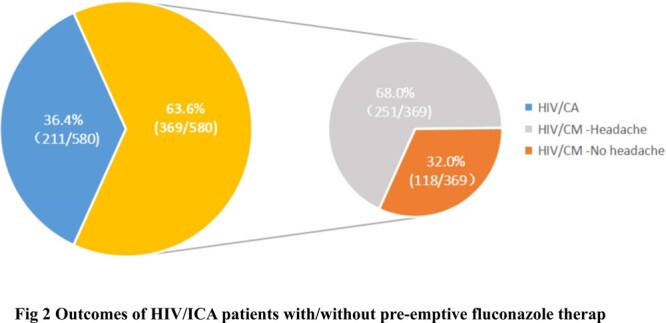

**Figure:**
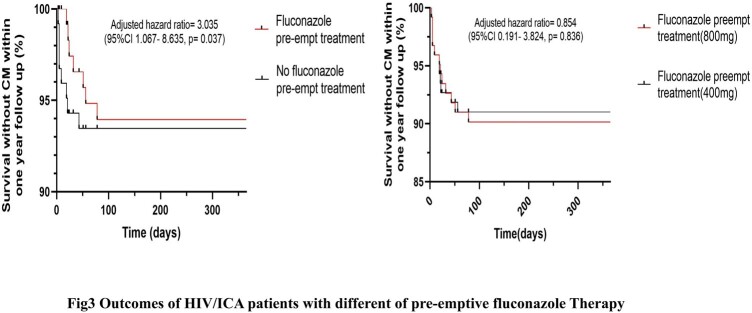

**Conclusion:**

Rapid ART initiation, appropriate CrAg screening are essential for advanced PLWH. Lp is a necessary intervention in advanced HIV-infected patients for CrAg positive individual to prevent potential missed diagnosis and mistreatment of CM. Targeted improvements to pre-emptive antifungal therapy for cryptococcal antigenemia are required to further improve patient outcomes in CrAg-positive severely immunocompromised people with HIV/AIDS.

**Disclosures:**

**All Authors**: No reported disclosures

